# SR Protein Kinase is expressed in *Drosophila* ovarian germline stem cells but is not essential for their self-renewal

**DOI:** 10.17912/micropub.biology.001550

**Published:** 2025-03-17

**Authors:** Victoria E. Garrido, William G. Outlaw, Amanda M. Powell, Elizabeth T. Ables

**Affiliations:** 1 Biology, East Carolina University, Greenville, North Carolina, United States

## Abstract

Germline stem cells (GSCs) are necessary for oocyte production in *Drosophila* . GSC maintenance is regulated by intrinsic factors that promote their self-renewal. One such factor, the beta-importin, *Transportin-Serine/Arginine rich* , mediates nuclear import of serine/arginine-rich (SR) proteins, which are phosphorylated by SR protein kinases. Here, we investigate whether the kinase encoded by *SR protein kinase* ( *SRPK* ) is essential for GSC self-renewal. We find that SRPK is expressed in GSCs and their mitotically-dividing daughters, but is not necessary for GSC establishment or maintenance. We conclude that SRPK is dispensable for GSC self-renewal, and postulate that other protein kinases can compensate for its absence.

**Figure 1. SRPK is widely expressed in the ovary, but dispensable for GSC maintenance:**
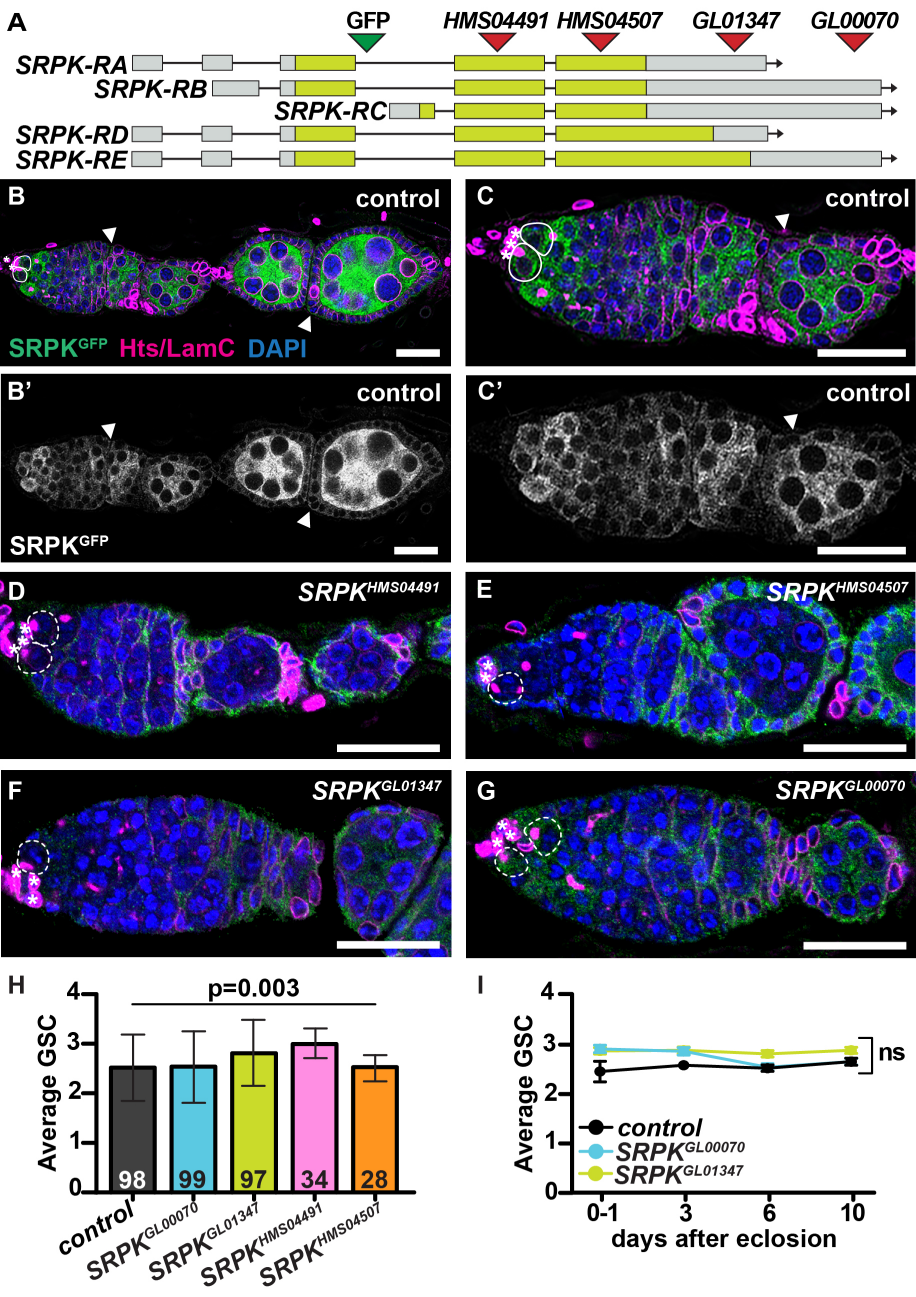
**A.** Schematic diagram of predicted *SRPK* mRNA isoforms. Triangles represent GFP insertion site (green) or regions targeted by RNAi hairpins (red). **B-G.** Ovaries from control *SRPK-GFP* females (B-C’) or *SRPK-GFP; nos-Gal4>SRPK ^RNAi^* females (D-G) labeled with anti-GFP (green; grayscale in B’-C’), anti-Hts and anti-LamC (magenta), and DAPI (blue). Panel C is magnified from panel B. Solid lines demarcate wild-type GSCs (white); dashed lines demarcate RNAi-expressing GSCs (white); asterisks mark cap cells; arrows point to follicle cells. Scale bars = 20µm (B, B’) or 10µm (C, C’-G). **H.** Average number of GSCs per germarium 6 days after eclosion, compared using one-way ANOVA. Numbers in bars indicate sample size. Error bars represent s.d. **I.** Average number of GSCs per germarium over a 10-day time course following eclosion, compared using a two-tailed Student’s t-test (n = >150 germaria per sample; see Methods). Error bars represent s.e.m.

## Description

Germline stem cells (GSCs) in the *Drosophila* ovary provide the cellular materials necessary to maintain oocyte production in adult females. Self-renewal of GSCs requires intrinsic factors that promote proliferation and asymmetric division, including those that regulate post-transcriptional processes such as RNA splicing and translation (Hinnant et al., 2020). One such protein, the beta importin, *Transportin-Serine/Arginine rich* (Tnpo-SR), is necessary for GSC self-renewal (Beachum et al., 2023). In somatic cells, Tnpo-SR facilitates intracellular transport of serine/arginine-rich (SR) proteins, a large family of pre-mRNA splicing factors containing an amino acid sequence rich in arginine/serine dipeptide repeats (RS domain) that also serves as a nuclear localization signal (Lai et al., 2001; Maertens et al., 2014). Tnpo-SR specifically recognizes phosphorylated SR proteins, influencing their subcellular localization (Lai et al., 2000; Lai et al., 2001). Whether phosphorylation influences Tnpo-SR cargo selection in GSCs is yet unknown.

To begin to assess relevant mechanisms, we asked whether phosphorylation of SR proteins is necessary for GSC self-renewal by investigating the role of SR protein kinase (SRPK), a non-specific serine/threonine kinase known to phosphorylate SR proteins at serine residues in the RS domain (Gui et al., 1994; Hogg and Findlay, 2023). SRPK is expressed in the later stages of oogenesis and is necessary for karyosome formation and meiotic spindle microtubule assembly in maturing oocytes, but has been poorly studied in the germarium, where GSCs and their mitotically dividing daughters are located (Hinnant et al., 2020; Loh et al., 2012). We identified an endogenously-tagged allele ( *SRPK ^MI06550-GFSTF.1^* ; henceforth referred to as *SRPK-GFP* ), in which green-fluorescent protein (GFP) was incorporated between the first and second coding exons, generating an in-frame fusion protein ( Figure 1A ; Nagarkar-Jaiswal et al., 2015). Using antibodies against GFP, Hu li tai shao (Hts; enables identification of GSCs and their dividing daughters by its expression in a germ cell-specific organelle called the fusome) (de Cuevas and Spradling, 1998), and LaminC (allows visualization of somatic cap cells adjacent to GSCs) (Ables and Drummond-Barbosa, 2013), we confirmed that *SRPK-GFP* is expressed in both germline and somatic cells throughout oogenesis ( Figure 1B ). It is highly expressed in GSCs and dividing cystoblasts, 2-cell, and 4-cell cysts ( Figure 1C ), with a significant increase in expression in differentiated germ cells once egg chambers have budded off from the germarium ( Figure 1B ). *SRPK-GFP* is also expressed in follicle cells at lower levels compared to its expression in the germline ( Figure 1B ’-C’). In all cells, SRPK-GFP localized exclusively to the cytoplasm, consistent with a potential role in phosphorylating cytoplasmic proteins for nuclear trafficking ( Figure 1B- C’).

Loss of *Tnpo-SR* decreased GSC establishment and maintenance, manifesting as decreased number of GSCs at eclosion and over the first ten days during adulthood (Beachum et al., 2023). We therefore hypothesized that if SRPK phosphorylation of Tnpo-SR cargo proteins is necessary for GSC establishment or maintenance, then loss of *SRPK* would similarly result in depletion of GSCs. To deplete *SRPK* specifically from GSCs, we used the germline-specific *nos-Gal4* in combination with expression of RNA hairpins using the UAS/Gal4 system (Blake et al., 2017; Ni et al., 2011; Van Doren et al., 1998). We identified three RNAi lines ( *SRPK ^HMS04491^* , *SRPK ^HMS04507^* , and *SRPK ^GL01347^* ) that effectively knocked-down *SRPK-GFP* expression in GSCs and their daughters ( Figure 1D- F), and one that decreased *SRPK-GFP* expression, but did not eliminate it ( *SRPK ^GL00070^* ; Figure 1G ). We then counted the number of GSCs per germarium in well-fed control ( *nos-Gal4* alone) and *nos-Gal4>SRPK ^RNAi^* females at six days after eclosion ( Figure 1H ). GSCs were identified by visualizing their anteriorly-localized fusome adjacent to cap cells using anti-Hts and anti-LaminC antibodies (Ables and Drummond-Barbosa, 2013). Although germarium structure and egg chamber morphology were normal, we found slightly higher numbers of GSCs in two *SRPK ^RNAi^* lines. To determine whether this slight increase in GSC number was biologically relevant, we expanded our analysis to count the number of GSCs over time from one to ten days after eclosion ( Figure 1I ). We selected two representative RNAi lines for the analysis, to compare the effects of either complete or partial knock-down ( *SRPK ^GL01347^* and *SRPK ^GL00070^* , respectively). In both cases, depletion of *SRPK* did not significantly impact the number of GSCs per germarium at eclosion, suggesting that *SRPK* is not necessary for GSC establishment during development. Further, we did not observe significant decline in GSCs in control or *SRPK ^RNAi^* germaria as females aged. These data suggest that depletion of *SRPK* does not inhibit GSC maintenance or continued division during adulthood.

In this study, we explored whether Tnpo-SR cargo proteins in GSCs are recognized by SRPK-dependent phosphorylation. We conclude that although SRPK is expressed in ovarian GSCs and their mitotically-dividing daughters, and is necessary for meiosis in differentiated oocytes (Loh et al., 2012), it is not essential for GSC establishment or maintenance. Importantly, our RNAi knock-down approach may not have completely eliminated *SRPK* expression; further, we did not investigate potential autonomous or non-autonomous roles of *SRPK* in somatic cells, where it is expressed (albeit at much lower levels). Nevertheless, our data complement a prior study by Loh and colleagues, who identified a *SRPK* mutant allele ( *SRPK ^129-09^* ) that yielded sterile, but otherwise normal, females (Loh et al., 2012). Consistent with our results, Loh and colleagues hypothesized that SRPK is specifically necessary for acentrosomal meiotic spindle assembly, but dispensable for mitotic spindle assembly. Taking these results together, we propose two possibilities. First, since SRPK is expressed in GSCs and mitotically-dividing germ cells, its loss of function could be compensated by other kinases. Two other SR kinases, *SRPK79D* and *CG8565* , are also encoded in the *Drosophila* genome, but mRNA levels of either gene are very low in ovarian cells (Li et al., 2022), making it unlikely that these can compensate for SRPK. However, in mammals, the cdc2-like kinase CLK1 can phosphorylate SRPK targets in its absence (Hogg and Findlay, 2023; Yeakley et al., 1999). One *Drosophila* CLK1 ortholog is *Darkener of apricot* ( *Doa* ), which is highly expressed in ovarian cells (Li et al., 2022). Doa is an essential Ser/Thr kinase that regulates microtubule binding, oocyte development, germ cell mitosis, and the mitosis-meiosis transition, and loss of *Doa* function produces phenotypes reminiscent of *Tnpo-SR* loss-of-function (Morris et al., 2003; Serpinskaya et al., 2014; Yun et al., 2000; Zhao et al., 2013). Thus, future studies should investigate potential genetic interactions between *Doa* and *Tnpo-SR* . Second, SRPK phosphorylation of target cargo may not be necessary for Tnpo-SR-dependent processes in GSCs. Although SR-rich splicing factors are the canonical substrates of SRPK and the primary cargo of Tnpo-SR, recent analysis of Tnpo-SR-cargo protein interactions *in vitro* suggest that it also binds proteins that lack canonical RS-domains (Kimura et al., 2021; Kimura et al., 2017). This may suggest roles for Tnpo-SR in centrosome function and/or cytoskeletal dynamics independent of nucleocytoplasmic shuttling of splicing factors (Kimura et al., 2017). Future studies exploring these two divergent possibilities will shed new light on how stem cells are maintained in adult tissues.

## Methods

***Drosophila* Lines and Husbandry**

Fly stocks were held at 22-25°C on standard cornmeal/molasses/yeast/agar medium (Genesee Scientific Nutri-Fly-MF). Fly lines used in this study are listed in the Reagents Table below. Flies were fed wet yeast paste plus standard media for three, six, and ten days prior to dissection (except newly eclosed flies, which were fed for one day prior to dissection).

**Immunofluorescence and Microscopy**

Immunofluorescence of whole ovaries was performed as previously described (Beachum et al., 2023). Briefly, ovaries were dissected and ovarioles teased apart in cold Grace’s insect media (Caisson Labs #GIL07) and transferred to a BSA coated tube on ice, then fixed for 13 minutes in 5.3% formaldehyde in Grace’s media at room temperature. Samples were washed extensively in phosphate-buffered saline (PBS, pH 7.4; ThermoFisher Scientific) with 0.1% Triton X-100 (PBS-T), permeabilized in 0.5% PBS-T for 30 minutes, and blocked for an hour in blocking solution [5% bovine serum albumin (Sigma), 5% normal goat serum (MP Biomedicals), and 0.1% Triton X-100 in PBS] at room temperature. Primary antibodies (see Reagents Table) were diluted in blocking solution and incubated overnight or over two nights at 4​°C: chicken anti-GFP (1:2000), mouse anti-Hts (1B1; 1:10), and mouse anti-LaminC (LamC; 1:100). Secondary antibodies (goat α-mouse-AlexaFluor568 or goat α-chicken-AlexaFluor488; ThermoFisher Scientific) were diluted in blocking solution and incubated for two hours or overnight at 4​°C. All ovary samples were stained with 0.5 ​μg/ml 4′-6-diamidino-2-phenylindole (DAPI; Sigma) in 0.1% Triton X-100 in PBS and mounted in 90% glycerol mixed with 20% n-propyl gallate (Sigma). Confocal z-stacks (1 ​μm optical sections) were collected with a Zeiss LSM700 laser scanning microscope using ZEN Black software. The images were analyzed using ZEN Blue and minimally and equally enhanced using Adobe Photoshop and Adobe Illustrator.

**Statistical analysis**

GSCs were identified by their anteriorly localized fusomes (Ables and Drummond-Barbosa, 2013). For initial screening, average GSC number was assessed in at least 28 random germaria from >10 females per sample. For time course analysis, the average GSC number per germarium for each genotype was assessed in over 150 germaria per sample in at least three independent experiments, each with >10 females per sample. Control and *SRPK* knock-down germaria were compared using one-way ANOVA (Prism) or two-tailed Student’s T-test (Prism), where * *p* <0.001 was considered statistically significant.

## Reagents

**Table:** 

***Drosophila* strains **			
**Strain**	**Genotype**		**Available From**
*SRPK-GFP*	y ^1^ w*; Mi{PT-GFSTF.1}SRPK ^M106550-GFSTF.1^	BDSC_65332	Bloomington *Drosophila* Stock Center (BDSC)
*nos-Gal4*	*nanos-Gal4::VP16*	BDSC_4937	BDSC
*UASp-LacZ*	P{w ^+mC^ =UASp-lacZ}1, w*	BDSC_98113	BDSC
* UAS-SRPK ^GL00070^*	y ^1^ sc* v ^1^ sev ^21^ ; P{y ^+t7.7^ v ^+t1.8^ =TRiP.GL00070}attP2	BDSC_35196	BDSC
* UAS-SRPK ^GL01347^*	y ^1^ sc* v ^1^ sev ^21^ ; P{y ^+t7.7^ v ^+t1.8^ =TRiP.GL01347}attP2	BDSC_42813	BDSC
* UAS-SRPK ^HMS04491^*	y ^1^ sc* v ^1^ sev ^21^ ; P{y ^+t7.7^ v ^+t1.8^ =TRiP.HMS04491}attP2	BDSC_57295	BDSC
* UAS-SRPK ^HMS04507^*	y ^1^ sc* v ^1^ sev ^21^ ; P{y ^+t7.7^ v ^+t1.8^ =TRiP.HMS04507}attP40	BDSC_57587	BDSC
**Antibodies**			
**Antibody**	**Animal and Clonality**		**Available From**
Hts	Mouse, monoclonal	AB_528070	Developmental Studies Hybridoma Bank (1B1)
LaminC	Mouse, monoclonal	AB_528339	Developmental Studies Hybridoma Bank (LC28.26)
GFP	Chicken, polyclonal	AB_300798	Abcam (#ab13970)
